# Perceived facilitators, needs, and barriers to health related quality of life in people with multiple sclerosis: a qualitative investigation

**DOI:** 10.1186/s41687-022-00496-1

**Published:** 2022-08-26

**Authors:** Erin Faraclas, Angela Merlo, Jeff Lynn, Jeffery D. Lau

**Affiliations:** 1 School of Physical Therapy, Massachusetts College of Pharmacy and Health Sciences, Worcester, MA USA; 2 Department of Physical Therapy, Eastern Washington University, Spokane, WA USA; 3 Slippery Rock University, Slippery Rock, PA USA; 4 College of Rehabilitation Sciences, Rocky Mountain University of Health Professions, Provo, UT USA

**Keywords:** Multiple sclerosis, Health and wellness, Quality of life, Lived experience

## Abstract

**Background:**

Multiple sclerosis (MS) is a chronic progressive neurological disease that influences an individual's physical, mental, emotional, and social functioning, otherwise known as health-related quality of life (HRQOL). To fully capture the impacts of MS on HRQOL, perspectives from the lived experience should be investigated.

**Objective:**

The purpose of this study was to describe, in people with relapsing–remitting multiple sclerosis (RRMS), (1) the health and wellness needs and facilitators perceived to influence HRQOL, (2) determine which health needs are not being met, and (3) identify barriers to meeting health and wellness needs.

**Methods:**

Participants with RRMS were recruited from a more extensive study for this cross-sectional, qualitative investigation guided by phenomenological theory. Semi-structured interviews were conducted until data saturation was reached (n = 15). The data were analyzed using a thematic analysis approach.

**Results:**

Five themes emerged as facilitators of HRQOL; mental/emotional health, knowledge about MS, family/peer support, lifestyle behaviors, and social engagement. Identified barriers to achieving better HRQOL included limited access to specialized care, lack of communication/ empathy from providers, lack of comprehensive care, challenges caused by MS symptoms, and difficulty navigating the healthcare and insurance landscape.

**Conclusions:**

Study participants described mental health and lifestyle behaviors as the primary promoters of overall HRQOL. Access to dietary guidelines, exercise instruction, and education about living healthy with MS were also identified as positive contributors to overall QOL. When these positive contributors are limited or absent, HRQOL was reported to decrease.

## Introduction

Multiple sclerosis (MS) is a chronic progressive neurological disease diagnosed commonly between 20 and 50 years of age, with over 2.3 million people living with MS worldwide [1, 2]. Approximately 85% of people diagnosed with MS initially present with relapsing–remitting MS (RRMS) [3–6]. RRMS is characterized by clearly defined periods of disease activity, known as exacerbations, followed by periods with partial to complete recovery of symptoms that repeats over time [3, 6, 7]. As a result of this pattern, individuals living with MS experience different health and wellness needs over their lives that directly influence their quality of life (QOL) [8].

Given the varied symptoms associated with MS, it is not surprising to see in the literature that individuals with MS have a decreased QOL compared to the general population [9–11]. In both 2008 and 2013, the Multiple Sclerosis International Federation (MSIF) and the World Health Organization (WHO) collaborated to develop the Atlas of MS, a document that identified knowledge gaps, resources, and services for individuals with MS [12]. The 2013 Atlas of MS, strongly suggested more research is needed at national, regional, and global levels to understand better factors that influence the QOL and experiences people with MS have [12].

Current literature still appears to be limited when considering the patients’ points of view and their identified health and wellness needs [13, 14]. One strategy to close this gap is to look at QOL from the lived experience perspective. A recent study examined the traffic on the National Multiple Sclerosis Society’s (NMSS) website and social media platforms to identify what content people coming to their web pages were interested in the most. The most frequently searched terms during the study period were diet, exercise, and emotional issues. This finding demonstrates that people with MS are interested in a vast array of different aspects of health and wellness [15].

To better understand the experiences of people with RRMS, the lived experience perspective needs to be included in the larger body of knowledge. Present literature routinely fails to consider patients’ points of view and their self-identified needs in the different health and wellness dimensions [13]. This raises the question, what do people with RRMS identify as essential for a high health related quality of life (HRQOL)? Likewise, what gaps or needs could facilitate improving the HRQOL in this population? Therefore, this study sought to explore the factors promoting HRQOL for people with RRMS through the lived experience.

The purpose of this study was to describe the perceptions related to health and wellness that individuals with RRMS identify as essential to their overall quality of life. The aims are to (1) identify and describe the health and wellness needs and facilitators perceived to influence HRQOL, (2) determine which health needs are not being met, and (3) identify barriers to meeting health and wellness needs.

## Materials and methods

This study used a cross-sectional, qualitative design guided by phenomenological theory [16]. This theory explores how individuals draw meaning from their experiences by describing the overall essence of an experience, the lived experience [17]. This study was conducted via telecommunication methods in the United States. Rocky Mountain University of Health Profession’s IRB approved this study.

### Participants

A purposeful sequential sample of convenience was used. The inclusion criteria were 18 years of age or older, self-reported diagnosis of RRMS, and ability to communicate by phone or video media platform for 30–60 min in the English language. Exclusion criteria included having a neurological diagnosis in addition to MS. The self-reported diagnosis of RRMS was on the honor system; participants did answer a few questions related to their MS diagnosis. These questions were reviewed by the principal investigator (PI).

### Recruitment

Participants were recruited from a group of 120 participants already enrolled in another larger study. The larger study surveyed 120 participants with RRMS comparing population-based normative data on the short form 36 (SF-36) to data collected from individuals with RRMS [18]. An invitation to participate in this second study was provided to all participants in the larger study. Purposive sequential sampling accounted for both sexes from those interested in participating in this study. The principal investigator conducted interviews until data saturation was reached (12 females, 3 males). Data saturation was determined to have been reached once no new themes or concepts arose in the interview for three consecutive participants. Participants ranged from 18 to 70 years old, with a mean of 44 years (SD 15). See Table 1 for demographic data.

**Table 1:** demographic data for study participants (n = 15)

	Mean/category	Range/frequency
Age	44.3 ± 15.1	18–70
Years with MS	13.3 ± 11.5	0–38
Sex	–	12F–3M
Employment	–	
	Full time	5
	Part-time	1
	Retired	2
	Unemployed	5
	College Student	2
Ambulatory status	–	–
	Independent	10
	Ambulatory device with 0-minimal limitations	3
	Ambulatory device with mod or more limitations	2

### Interview

Each participant completed one semi-structured interview lasting an average of 38 min (range = 27–65 min). All participants were interviewed over the phone. All interviews were recorded with participant consent.

The semi-structured interview guide (see “Appendix”) focused on exploring the different domains of health perceived to impact QOL. Contextual details related to what was essential to improve or maintain a high HRQOL, living with RRMS was emphasized. At the beginning of the interview, sociodemographic data were collected (i.e., age, sex, years living with MS, employment status, insurance coverage, income, and ambulatory ability). The final interview question asked participants to review a list of eight health domains of health and identify the area(s) they felt were most important to their overall QOL. Participants were provided an Amazon gift card of $25.00 for participating in the interview.

## Data management and analysis

All fifteen interviews were recorded via a digital recording device and transcribed verbatim by a secure transcription service. All recordings were compared to the transcript to ensure accuracy. Transcripts were sent to each study participant for a member check, and each participant was asked to check the transcript for accuracy. Once all member checks were complete, the interview transcripts were uploaded into Nvivo 12 data analysis software (QSR International Pty Ltd., Burlington, MA).

The principal investigator reviewed transcripts multiple times before coding the data set [19, 20]. Data was coded using inductive coding [20] and analyzed using a thematic analysis approach. Inductive coding is a commonly used approach to qualitative research in the health sciences [21]. Inductive coding codes come from the data itself versus deductive coding, which uses theoretical or epistemological approaches. Boyatzis first described this inductive method of data-driven coding [22]. Thematic analysis with inductive coding identifies, analyzes, and reports themes that emerge within the data set [19, 23]. Once all codes were identified, themes were derived from the coded data. A detailed review of the codes and themes was conducted, and revisions occurred as appropriate.

Several trustworthiness strategies were used throughout the study. Member checks for accuracy of the transcribed data occurred before the coding process began. A peer reviewer experienced in qualitative data examined the coded transcripts and reviewed all major themes, and the principal investigator maintained an audit trail. Both researchers met to discuss the data. The principal investigator then refined the codes and major themes. The principal investigator recorded the data analysis process to record the decision-making process. A reflexivity journal to identify for and control biases was maintained as well.

## Results

Of 15 participants, twelve were female, and three were male (80% women, 20% men). These percentages are similar to the general MS population in the United States [1]. The mean age of the participants was 44.3 years, SD ± 15.1. Years with MS ranged from one year to 38 years. The mean length of time since diagnosis was 13.3 years, SD ± 11.5. Some participants no longer worked secondary to MS, while others were employed full-time. Two participants were full-time college students. The participants for this study resided in varied geographic areas providing a good representation of the United States. The participants lived in urban, suburban, and rural areas throughout the United States.

**Fig. 1:**
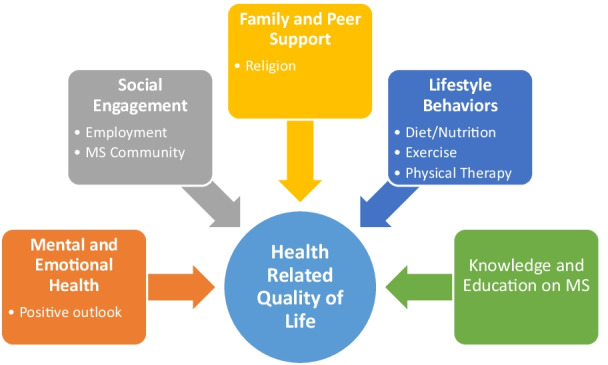
Promotors of health related quality of life

**Fig. 2:**
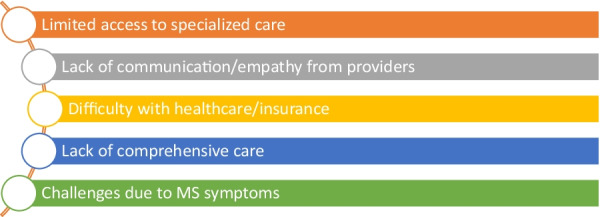
Barriers to Health

Five clearly defined themes emerged as promoters of HRQOL (Fig. 1). These themes are mental/emotional health, knowledge/education about MS, family and peer support, lifestyle behaviors, and social engagement. Additionally, five main barriers to achieving better health and quality of life were present in the data (Fig. 2). These barriers included limited access to specialized care, lack of communication and empathy from medical providers, lack of comprehensive care addressing all health-related needs, challenges caused by MS symptoms, and difficulty navigating the healthcare and insurance landscape. Lastly, participants were asked to look at the list of eight domains of health as reported on the short form 36 (SF-36) to identify the domains of health that are essential to have a good QOL living with MS and to list the top three domains that are the most important to live your best life with MS. Mental health, role emotional, and social function were the most frequently cited domains, see Table 2. Table 2 lists the frequency of the items identified by the 15 participants. In the second column, *Top 3 Most Important to Live Best Life*, all participants were required to report 3 items, for a total of 45 reported items. In the first column, participants were allowed to report variably between 1 and 3 items, where a total of 33 reported items were collected.

**Table 2:** Top dimensions of health reported as essential for improving HRQOL

	Frequency—essential to have a good HRQoL	Frequency—top 3 most important to live best life
Physical function	4	5
Mental health	10	14
Role physical	2	3
Bodily pain		
General health	1	2
Vitality		2
Social function	5	9
Role emotional	11	10
Total	33* participants reported 1–3 items	Total 45* all participants listed 3

### Promotors of health related quality of life

#### Mental/emotional health

This study identified mental and emotional health as the most important facilitator of their HRQOL. Nearly all participants (13/15) discussed mental and emotional health as having a considerable influence on their overall HRQOL. Identifying, diagnosing, and addressing mental/emotional health concerns were the most frequently discussed theme when asked about what is needed to live healthily and well with MS. The top three identified HRQOL promoters were mental and emotional health, lifestyle behaviors (diet, exercise), and family and peer support. One participant stated, “MS affects all parts of your brain, which affects not just physical, but mental and emotional health too. Making sure to address that, so your whole self is better, is important.”

Eight participants reported suffering from depression, anxiety, or other mental/emotional health problems throughout their MS disease course, representing over 50% of the study participants. Only two participants of the eight reported ever receiving any intervention for their mental health needs. Four of the six participants who did not receive mental health interventions expressed a strong desire to discuss mental and emotional health with their healthcare providers. “I want to be mentally healthy,” stated one participant, commenting that no provider has asked her about mental health, and no support has ever been offered. Another participant indicated her QOL is not as good as it could be because of her mental health, and she voiced how beneficial it would be to have a provider address these needs. Conversely, mental and emotional health was also identified as the greatest need, yet the area reported to be mentioned the least. The barriers to improving mental and emotional health were described as lack of comprehensive care, poor healthcare coverage, and lack of compassionate and caring providers.

Fourteen out of fifteen participants reported having a positive outlook and attitude as a strong promoter of a good HRQOL. Optimistic views, never giving up hope, learning about MS, and staying positive were essential for living well with MS. This positive outlook was also a desired attribute from providers; study participants wanted to see a positive attitude in their healthcare providers, family, community, and friends.

#### Social engagement

According to 14/15 participants, having peer support, positive social interaction with peers or other individuals with MS, and close personal relationships were necessary to have a good QOL. Participants spent significant time talking about their social circle, or lack thereof, and the importance of social engagement to their overall wellbeing. The college-age participants described the impact that a lack of social interactions has on the individual. “Universities could do better at providing opportunities for disabled students,” reported one college-age participant. Both college students verbalized a strong desire to stay engaged and interact with peers and their college community.

“For me, one of my big difficulties with MS was social because I had just started college with this diagnosis and with I guess the fatigue issues I had, I could not go to the football games and the basketball games, and so I guess it is more just extra social things, that are important to my mental health, but at college, these social [things] are not easy to do.”

Five other participants cited the fear of social isolation as a reoccurring concern and detractor from HRQOL. One study participant stated, “I really do not have that much of a support system, and it is isolating.” Participants shared experiences of feeling isolated due to common MS symptoms and lack of peer or family support. Connecting with others who have MS and therefore understanding the challenges of MS was reported to help mitigate social isolation fears. “I think when you are talking to other people who have had MS– especially if you are newly diagnosed; they know what you are going through, and it helps.” Receiving education from peers about MS was frequently cited as a strong promoter of a good HRQOL, especially for those newly diagnosed.

Employment status was another identified promoter of a good HRQOL linked to social engagement. Individuals in the workforce cited working as a positive influencer for their current HRQOL, tying in the workplace with social engagement. “I have always worked, and thankfully I have always been able to work,” said one individual. He further explained the benefits he received from working, such as providing for his family, staying socially engaged, and physically active. Participants who were unable to work reported social isolation, financial and insurance problems, and emotional health concerns resulting from unemployment.

When participants reported barriers or areas of need to improve HRQOL, most participants linked decreased social roles or community engagement causing an overall negative effect on their QOL. Like mental and emotional health needs, social needs were ranked high on the HRQOL promotors but also near the top of domains in health that need to improve to facilitate better HRQOL.

#### Family and peer support

Family and peer support was another theme expressed as a strong facilitator for HRQOL. All fifteen participants communicated the positive benefits of family and peer support throughout the interview. Neighbors, friends, coworkers, and church members were described as providing physical, mental, social, financial, and educational support. This support increased positivity and a sense of purpose, ultimately leading to a greater perceived QOL. “A strong support system from a lot of different places,” voiced one participant when asked about facilitators of good health. One participant stated, “I found out the hard part with MS is it can be very hidden, and people do not see your pain or your weakness until you are showing symptoms.” She further stated how beneficial having a support system can be because of these “hidden” symptoms. When pressed to explain this further, she explained that support systems understand you and your MS, which is not always the case with people I do not know.

Engaging in the MS community for 12 participants was another critical support system. One participant stated, “I am a member of a group of moms with MS….and that kind of helps just to feel like I am not alone; I am not crazy going through all this.” Three additional participants specifically cited connecting with others who had MS as emotionally beneficial. Although family and peers were ranked as most important in supporting the participants, twelve participants cited the MS community as an additional and significant support source. One participant currently navigating college articulated, “when you are talking to other people who have it [MS], they know what you are going through.” She expressed a deep desire to connect with others who understand the lived experience with MS. Another participant, also in college, reported that getting involved in the MS community led to her receiving a scholarship from the NMSS that helped her stay in college; when MS was causing “problems.” Some participants became involved in even more structured MS communities as well. “I have actually been leading and continue to lead a self-help group for Latinas with MS,” stated one middle-aged participant. She continued to explain how her involvement in the MS community keeps her feeling engaged and vibrant despite MS.

Spirituality and religion were also frequently brought up when discussing support systems. Several participants reported spirituality, a relationship with God, or their church group as essential to their overall QOL. The religious community for them was described as a strong sense of social interaction and support they could rely on.

“I think that MS has strengthened my relationship with God. I am a spiritual person, and so I have faith that God will hear my prayers and that if I do my part, I'll be blessed, and he'll help me overcome these challenges that come my way related to MS.”

#### Lifestyle behaviors

Another prominent theme that participants discussed frequently was diet, nutrition, exercise, physical therapy, and general wellness strategies. Participants reported that to promote healthy living with MS, they realized lifestyle behaviors such as diet and exercise were essential to improve their overall HRQOL. Fourteen participants reported exercise and physical activity as essential components of their own health promotion. “Exercise, I think, is extremely important,” stated one participant. He further expressed how exercise has prevented him from “succumbing” to some of the MS symptoms he has experienced. Walking and general physical activity were explicitly reported as improving mental and emotional health, QOL, overall improved health, and physical function. “I think that being able to exercise no matter what, whether it is a stationary bike or whether it is whatever you love to do, yoga or something, I think that is huge for a person with MS, to find something that can keep them physically strong and emotionally well,” stated a participant.

Participants defined physical activity as staying active, engaging with family and peers, maintaining employment, participating in hobbies, and accessing their community. Participants detailed how physical activity allowed for meaningful experiences with their family, community, children, and peers. One participant stated, “exercise can lead to a social life.” Fourteen participants reported exercise, yoga, or Pilates as beneficial promoters of overall HRQOL. “I am not the kind of person that loves exercise. I have to really push myself to do it. But I do feel better when I do it, and I know that improves my quality of life.” Another participant reported, “I have been doing yoga off and on for 17 years, and I modify as needed, but it really helps me move better”. All participants expressed that healthcare providers' emphasis and guidance on exercise and physical activity would improve their HRQOL. Despite the desire for information on exercise, participants only reported exercise guidance if they received physical therapy services. Participants also linked lower HRQOL to a lack of diet and exercise knowledge. Commonly lack of understanding of essential diet, exercise, and stress management strategies was perceived as an area of need to improve overall HRQOL. Barriers to lifestyle needs were deeply rooted in the feeling that medical care lacked comprehensive care and providers were only focusing on medical and physical symptoms of MS.

Nine of the fifteen participants reported diet, nutrition, and or supplements as strategies they specifically use to improve their health and wellness and overall HRQOL. Eight of the nine participants indicated they investigated and implemented diet strategies independently versus receiving guidance from their healthcare providers. “You want that education piece to be there, not just the pharmacological management but diet and nutrition or other ways to also be healthy.” The participant articulated that not enough value was placed on diet and lifestyle behaviors during her medical visits. Diet was one of the most frequently cited themes participants wanted to see improvements made to enhance their HRQOL. One male participant summed it up, stating,

“MS completely kind of changed my lifestyle completely changed my diet. I was already a very active individual but began making exercise more of a daily routine. So I have been able to, I feel like, manage my symptoms over the past almost 11 years now with diet, exercise, and then stress management. I try to get some sunshine, spend as much time in the sun as I can too.”

Five participants reported that physical therapy improved their HRQOL, and all five reported receiving an exercise plan that helped improve their mobility. These participants indicated they would like physical therapy or access to a professional who can more routinely assist with an exercise program to improve their current QOL. The other 10 participants reported that their providers never brought up exercise or physical therapy. Feelings of uncertainty about exercise were routinely reported as obstacles to improving their HRQOL. “I would love for somebody to just draw out an exercise plan for me.” This young female participant continued to discuss how this exercise plan would benefit her physical, mental, and overall health.

#### Knowledge and education on multiple sclerosis

Receiving patient-centered education with credible information about living healthy and well with MS was voiced by all participants as essential to HRQOL. Participants reported that education from their providers and the National MS Society (NMSS) positively influenced their ability to live well with MS. “I think it is really empowering to have all that knowledge and information,” said one female. The most commonly expressed need for increased education was related to exacerbations, fatigue management, stress reduction, cognitive changes, mental health, lifestyle behaviors, and general MS strategies for heat sensitivity. Participants in this study wanted more information on MS and for that information to come from their MS clinic or MS providers. Participants described how they gathered information from different sources and often felt overwhelmed or confused when determining what was accurate information on MS. When presented with good quality information; participants discussed how that information assisted them in improving or maintaining a high HRQOL.

### Perceived quality of life barriers

Throughout the interviews, participants frequently portrayed discrepancies between what they reported as essential for their health versus what they were currently receiving for healthcare. This group reported limited access to certain types of care such as mental health, instruction on lifestyle behaviors (diet, exercise), and accessing their community. They also described barriers related to social isolation concerns, challenges due to MS symptoms, difficulty navigating the healthcare and insurance landscape, and poor communication and empathy from healthcare providers. This group of individuals felt that specialized providers' education and information related to MS would significantly elevate their QOL.

While every participant discussed physical symptoms associated with MS and its impact on HRQOL, the physical health domain was not what the conversation centered around. Instead, participants highlighted health aspects outside the physical dimension of health as most influential to their HRQOL. Participants described unique and specific needs to their situation and MS presentation. All fifteen participants reported barriers to better HRQOL related to five common reasons: lack of MS-specific knowledge, limited access to specialized care, limited communication and empathy from their providers, lack of comprehensive care, and difficulty navigating the healthcare and insurance landscape.

Thirteen of fifteen individuals reported their healthcare providers did not discuss lifestyle factors such as diet and exercise. “Education about nutrition [is important] because there seems to be confusion about what is good for MS, simple classes for people to learn about diet would be a big help,” stated one middle-aged female when discussing her desire for more lifestyle information from her providers. Most participants saw this lack of dialog as a significant obstacle to achieving better health. “I am trying to avoid any other health issues; just having a good general well-being with exercise is tough but very important.” Although several participants mentioned vitamins and supplements throughout the interviews as strategies to stay healthy, participants did not mention their disease-modifying MS medication. Participants did not discuss medications at all. Participants instead focused their attention on other aspects of health during these interviews. The only participant to mention medication brought up medication to demonstrate that medicationss should be included in her treatments.

*“I have a lot of lesions up at my brain stem, which causes nystagmus and bladder issues. I have some bladder issues that I have had for a while. But I see a urologist, so they have got me on medication. The leg weakness-- I started taking the Ampyra for and special glasses for nystagmus. Diet and exercise would also help me, wouldn’t it?”*

Participants reported mental health as the area of health most in need when asked what they specifically need to improve their HRQOL living with MS. “I just feel like perhaps I can get more balance. I think, to me, wellness is balance. And right now, I really do not have much [due to MS]”, said one participant in her fifties. Others voiced that even when referred to therapy or counseling, the provider was not knowledgeable about MS, creating a poor experience. “They offered me counseling, but they [counselor] had no idea what MS was, and that was a problem,” one participant said.

Throughout the interviews, it became apparent that some barriers were due to their location. Several participants reported living too far from specialized providers and noted less than excellent care from providers not specialized in MS. One participant only sees his primary care physician for all his health needs because the closest MS specialist is over 4 h away. Other participants reported having an excellent specialist and attributed their overall success and HRQOL to that specialized care and provider. One recurrent detracter of health was the time between office visits with providers. Participants voiced a thirst for knowledge and more engagement from healthcare providers.

*“I want to talk to others who have MS or healthcare professionals that have dealt with MS and just be completely open. I feel like there is-- even with incontinence and stuff, there is still kind of-- people do not want to talk about it. So I would love an openness in a dialog between a healthcare provider and me. I feel like healthcare providers do not ask the right questions.”*

More than half of the participants raised concerns about adequate health insurance coverage for specialized providers. Even when insurance coverage was available, participants reported problems obtaining the care because the process was too complicated or confusing. One participant noted, “It was like a full-time job, going through the right hoops or the right process with the right forms.”

All fifteen participants were eager for information and guidance related to lifestyle behaviors such as diet and exercise. Participants expressed a desire to improve their HRQOL and live healthy despite MS. One participant remarked how a lack of information on diet and exercise was a “lost opportunity” for providers. He further described how diet, exercise, and sleep were his primary means of maintaining a high HRQOL. He would like to see these aspects of health and QOL integrated more into overall MS care from all providers. Other participants described a feeling of uncertainty in proceeding with exercise or nutritional changes to support their MS due to lack of knowledge. Despite this uncertainty about implementing lifestyle changes, the participants all seemed to coalesce around the idea that diet and exercise can facilitate improved HRQOL with MS.

Five of the fifteen participants characterized the facility they access as a comprehensive MS center. These individuals discussed their center's additional services, including physical therapy, education classes, counseling, and support groups, as valuable to their overall healthcare and QOL. Participants voiced the benefits of having a broader approach to their MS and cited the ease of communicating with the care team as instrumental in staying healthy. In contrast, a participant outside this group of five stated, “I feel that I am not getting support from the healthcare providers.” He explained that he needed more than just biannual visits and pharmacological management.

## Discussion

People with RRMS view health and HRQOL as a combination of different lifestyle behaviors, specialized care, and advanced knowledge of MS. The perspectives from the lived experience illustrate how these multiple factors interact and influence HRQOL. All participants in this study viewed HRQOL as a culmination of physical, mental, and social health. Although discussion about physical function was present in the interview data and had apparent effects on HRQOL, physical function was not perceived as most in need of improvement. Mental, emotional, and social health constructs dominated the conversation when asked “what keeps you healthy and well” and when asked about “barriers to achieving better health.”

Mental and emotional health was reported as having the greatest impact on HRQOL. The data showed that people with MS are asking for significantly more attention to mental and emotional health. Those findings are consistent with previous research investigating similar constructs [24–27]. A recent cross-sectional study demonstrated that people with MS have a decreased quality of life in all health domains on the short form 36 quality of life scale (SF-36) compared to the general population [18]. Mental, emotional, and social health domains on the SF-36 were the furthest below the population-based norms than other domains such as physical function [18]. This perspective of focusing on non-physical domains of health was also seen in this qualitative study. Only a few participants reported having mental health issues discussed or addressed by healthcare providers, yet almost all participants expressed a desire to have this support available. This discrepancy between what people with MS perceive as needed versus what is provided continues to persist today [24, 26, 28].

In addition to mental and emotional health, lifestyle behaviors such as diet and exercise were emphasized as essential to maintain or improve HRQOL. Current literature has suggested that focusing on patient perceptions and goals for living healthy with MS should further advance the patient-centered care model [29]. Despite the call for action on improving lifestyle behaviors and education about lifestyle behaviors for people with MS, we still see that implementing these practices is lacking [11, 15].

During the interviews, participants did not discuss, bring up, or report medication-related health needs. This omission may be due to the broad scope of health-promoting behaviors that are of more interest to the person since their primary MS medication needs are being addressed and the primary focus of most medical appointments [30]. Disease-modifying medications are vital to slow the disease down and decrease exacerbations; significant strides have been made over the last 20 years related to medications [31]. This past focus on medication may explain why participants in this study focused their attention on the nonpharmacological interventions to improve their health.

When participants discussed their healthcare providers, they stressed that knowledgeable and compassionate providers are essential to their HRQOL, especially when newly diagnosed. The healthcare provider was described as an essential component of managing MS. Providers were viewed as facilitators of good health, neutral (no impact) on health, or a detractor to health. Detracters were described as lacking compassion, not current on MS care, or failing to address all MS needs. These findings that healthcare providers need to be more empathetic and compassionate are consistent with existing literature [26]. Participants in this study described empathy and compassion as critical for establishing trust and rapport between patient and provider.

Having a robust support system is also essential to overall HRQOL. Social and community engagement was discussed more than expected throughout the interviews. Social isolation and limited engagement in society due to MS symptoms were common fears and detractors for HRQOL. Despite this perceived importance, participants did not see this level of importance emphasized or mentioned by their healthcare providers.

## Conclusion

Data from this study suggests opportunities to advance the HRQOL for people with RRMS are plentiful. All individuals with MS should be screened for mental or emotional health concerns. Questions and discussions should occur related to social engagement, and the person’s ability to engage with their family, community, work or church should be included explicitly in routine patient encounters. Screening for and including intervention strategies for lifestyle behaviors like diet, exercise, and stress management continue to be requested by the MS population. Despite numerous calls from patients with MS to incorporate more lifestyle behavior education into their treatment plans, people with MS still report that level of care is lacking.

These findings suggest that mental and emotional health influence HRQOL, and these are the same areas reported to have the greatest need for improvement. The most frequently reported health need was access to mental healthcare. These findings echo previous research done in other countries around the world [9, 26, 27, 32].

The lived MS experience is rich with meaningful information to help advance the HRQOL for this population. More studies need to be conducted looking at the perceptions and experiences of people with MS, what HRQOL means to individuals, and how to incorporate more health domains into the standard of care. Individuals in this study were clear in their message: mental health and lifestyle behaviors are paramount to their HRQOL, yet these are the health domains most under-supported in the healthcare they receive. Attention to the discrepancy in what individuals with RRMS report they need versus what they receive must continue to be investigated. Integrating lifestyle behaviors, screening questions for mental health, and social isolation should be integrated into all routine visits, especially now that we understand these factors' impact on overall HRQOL [33].

Barriers to better health continue to be complex and individualized, yet despite that, we continue to see common barriers within the control of healthcare providers. Participants in this study expressed the need for more compassionate and empathetic providers that listen to all the impacts they are experiencing due to MS. This sentiment is found throughout the literature related to MS and other chronic diseases [13, 34, 35]. From the lived experience perspective, compassion and empathy facilitate strong patient-provider relationships.

Several factors limit the generalization of the study findings. The sample included fifteen participants living in the United States. Nine different states were represented in this study population, which may not reflect other regions of the United States or worldwide. Recruitment for this study was limited to those participating in the more extensive MS study. This study also relied on a self-reported diagnosis of RRMS that their medical provider did not confirm, making it possible that the self-reported diagnosis is inaccurate. Future studies should recruit from different geographic regions in the United States, have an MD confirmed diagnosis of RRMS, and have a larger sample size. The principal investigator was the only one to analyze the data. Although a peer reviewer reviewed all significant codes and themes identified in the study, bias is possible from having only one reviewer. Another limitation of this study was that participants were not asked if they were currently in an exacerbation; this would be helpful information to gather in the future.

In summary, individuals with RRMS describe their HRQOL as a reflection of their health and wellness in multiple health domains. These findings suggest that individuals with RRMS seek guidance and expertise in lifestyle behaviors like diet and exercise. These same participants place a high value on mental and emotional health and desire healthcare providers to identify and address not just physical symptoms of MS but also mental and emotional symptoms. According to this study’s data, increased attention to mental health and access to mental health services will positively influence HRQOL along with education and interventions targeted at diet, exercise, and stress management.

## Data Availability

All de-identified data is available upon request.

## Appendix

### Interview guide

1. I thought we could start by having you describe what health and wellness mean to you.
  - Why do you think that is important for health and wellness?
2. What would you say is important for you to be healthy and well living with MS? Please describe your current health and wellness needs as related to your MS.
  - *Why are these (insert need(S) described by the participant) critical to you and your personal health and wellness?*
    (i) Can you explain a little more about why this need is essential to you?
    (ii) How does this influence your overall health and wellness?
  - *So you feel (insert the why from above) is important to you, are there other needs or aspects of your health and wellness that are important to you? Please describe this need for me.*
3. Please share how the needs you just described relate to your multiple sclerosis?
  - *How do these needs influence your overall health and wellness living with MS?*
  - *Please share with me how you feel these health and wellness needs are being met? (satisfactory, minimally, not met?)*
4. Can you describe any aspects of your health and wellness that are not fully supported?
  - *Why do you feel this is important to your health and wellness*
    (i) *Have you explored strategies to address this need?*
    (ii) *Why do you feel this strategy was or was not successful?*
5. What do you feel is needed to fulfill this unmet need? / What is limiting you from fulfilling this need?
  - *Do you perceive any barriers limiting your ability to meet this need? Please explain.*
6. Looking at this list of dimensions of health and wellness, are there any you feel are essential to having a good quality of life living with MS?
  (i) Physical Function
  (ii) Mental Health
  (iii) Role Physical
  (iv) Bodily Pain
  (v) General Health
  (vi) Vitality
  (vii) Social Function
  (viii) Role Emotional
  (ix) *How does (insert the dimension selected above) help you manage your MS and live well?*
  (x) *Looking at this list, would you say the three most important dimensions of health to you are to live your best life?*
    (i) Physical Function
    (ii) Mental Health
    (iii) Role Physical
    (iv) Bodily Pain
    (v) General Health
    (vi) Vitality
    (vii) Social Function
    (viii) Role Emotional
7. Do you have any additional information you would like to share?
  - *If there were areas to explore, then further that would occur here*
  - *If a question was left unanswered, it could be asked here.*
  - *I would end with a summarizing statement(s), allowing the participant to correct and/or verify that I had an accurate interview summary.*
